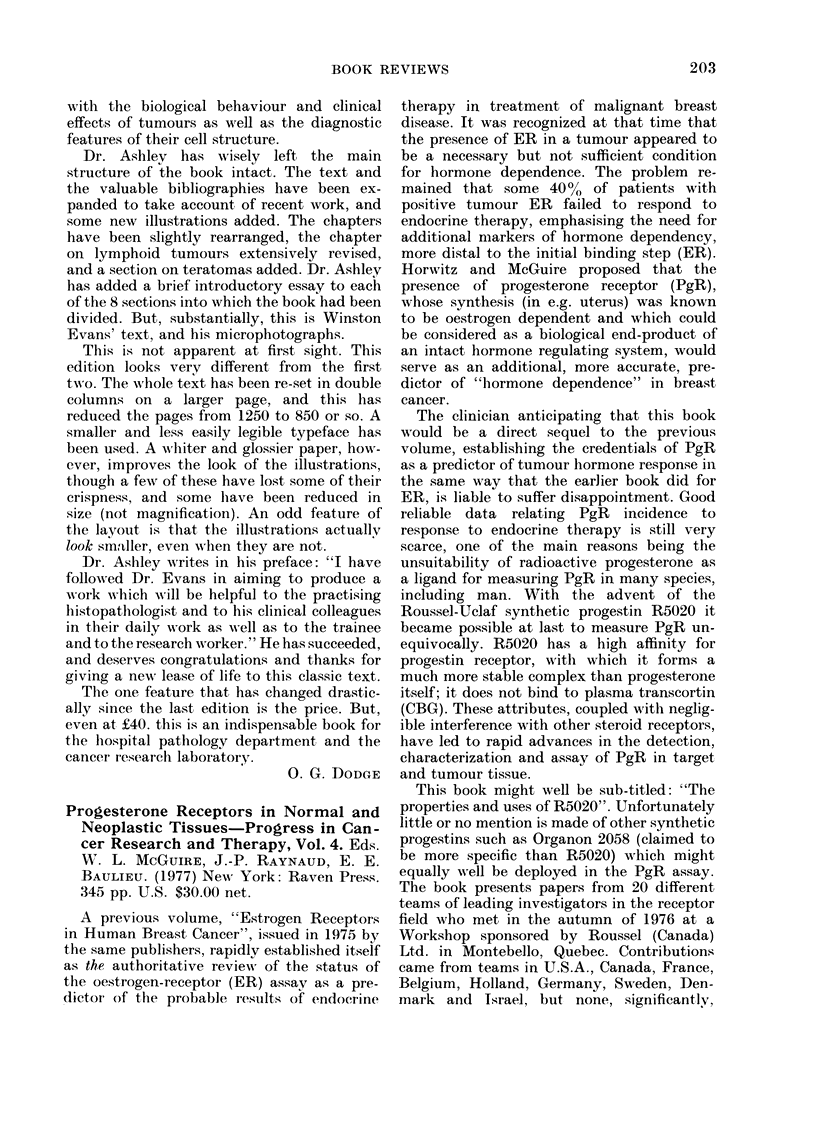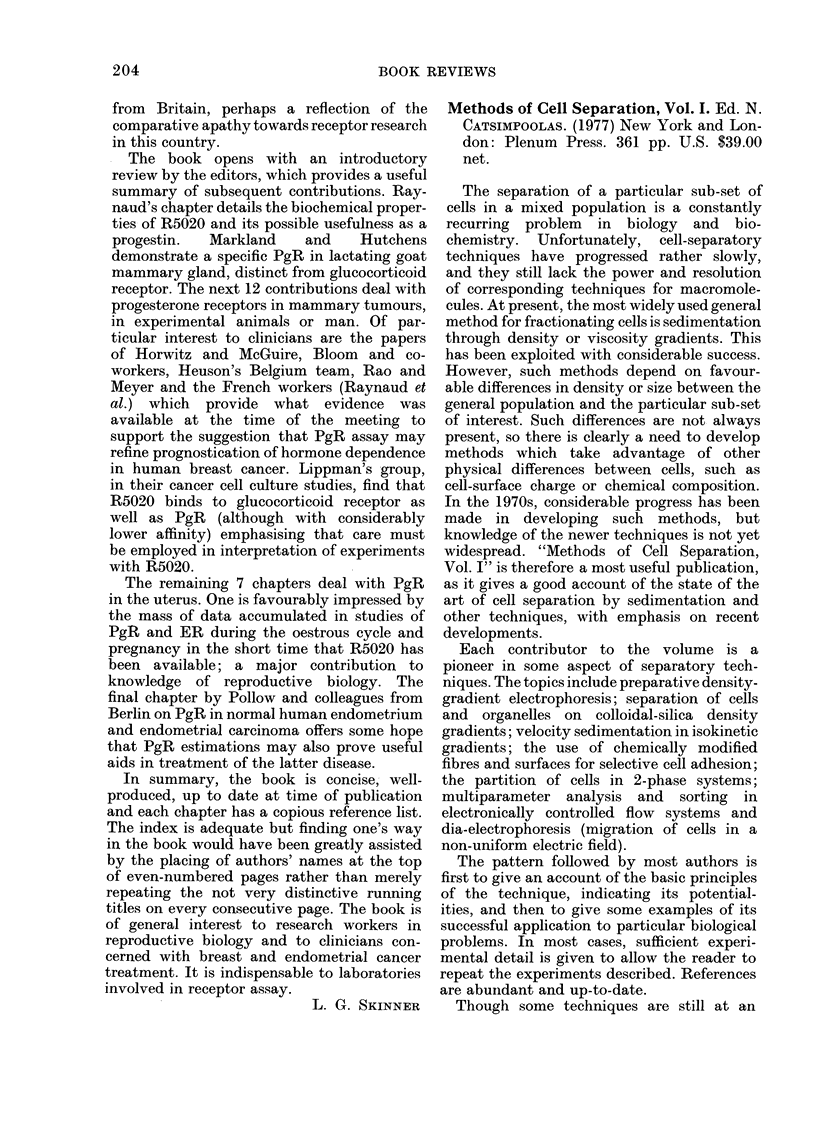# Progesterone Receptors in Normal and Neoplastic Tissues—Progress in Cancer Research and Therapy, Vol. 4

**Published:** 1978-07

**Authors:** L. G. Skinner


**Progesterone Receptors in Normal and Neoplastic Tissues—Progress in Cancer Research and Therapy, Vol. 4.** Eds. W. L. MCGUIRE, J.-P. RAYNAUD, E. E. BAULIEU. (1977) New York: Raven Press. 345 pp. U.S. $30.00 net.

A previous volume, "Estrogen Receptors in Human Breast Cancer", issued in 1975 by the same publishers, rapidly established itself as *the* authoritative review of the status of the oestrogen-receptor (ER) assay as a predictor of the probable results of endocrine therapy in treatment of malignant breast disease. It was recognized at that time that the presence of ER in a tumour appeared to be a necessary but not sufficient condition for hormone dependence. The problem remained that some 40% of patients with positive tumour ER failed to respond to endocrine therapy, emphasising the need for additional markers of hormone dependency, more distal to the initial binding step (ER). Horwitz and McGuire proposed that the presence of progesterone receptor (PgR), whose synthesis (in e.g. uterus) was known to be oestrogen dependent and which could be considered as a biological end-product of an intact hormone regulating system, would serve as an additional, more accurate, predictor of "hormone dependence" in breast cancer.

The clinician anticipating that this book would be a direct sequel to the previous volume, establishing the credentials of PgR as a predictor of tumour hormone response in the same way that the earlier book did for ER, is liable to suffer disappointment. Good reliable data relating PgR incidence to response to endocrine therapy is still very scarce, one of the main reasons being the unsuitability of radioactive progesterone as a ligand for measuring PgR in many species, including man. With the advent of the Roussel-Uclaf synthetic progestin R5020 it became possible at last to measure PgR unequivocally. R5020 has a high affinity for progestin receptor, with which it forms a much more stable complex than progesterone itself; it does not bind to plasma transcortin (CBG). These attributes, coupled with negligible interference with other steroid receptors, have led to rapid advances in the detection, characterization and assay of PgR in target and tumour tissue.

This book might well be sub-titled: "The properties and uses of R5020". Unfortunately little or no mention is made of other synthetic progestins such as Organon 2058 (claimed to be more specific than R5020) which might equally well be deployed in the PgR assay. The book presents papers from 20 different teams of leading investigators in the receptor field who met in the autumn of 1976 at a Workshop sponsored by Roussel (Canada) Ltd. in Montebello, Quebec. Contributions came from teams in U.S.A., Canada, France, Belgium, Holland, Germany, Sweden, Denmark and Israel, but none, significantly, from Britain, perhaps a reflection of the comparative apathy towards receptor research in this country.

The book opens with an introductory review by the editors, which provides a useful summary of subsequent contributions. Raynaud's chapter details the biochemical properties of R5020 and its possible usefulness as a progestin. Markland and Hutchens demonstrate a specific PgR in lactating goat mammary gland, distinct from glucocorticoid receptor. The next 12 contributions deal with progesterone receptors in mammary tumours, in experimental animals or man. Of particular interest to clinicians are the papers of Horwitz and McGuire, Bloom and coworkers, Heuson's Belgium team, Rao and Meyer and the French workers (Raynaud *et al.*) which provide what evidence was available at the time of the meeting to support the suggestion that PgR assay may refine prognostication of hormone dependence in human breast cancer. Lippman's group, in their cancer cell culture studies, find that R5020 binds to glucocorticoid receptor as well as PgR (although with considerably lower affinity) emphasising that care must be employed in interpretation of experiments with R5020.

The remaining 7 chapters deal with PgR in the uterus. One is favourably impressed by the mass of data accumulated in studies of PgR and ER during the oestrous cycle and pregnancy in the short time that R5020 has been available; a major contribution to knowledge of reproductive biology. The final chapter by Pollow and colleagues from Berlin on PgR in normal human endometrium and endometrial carcinoma offers some hope that PgR estimations may also prove useful aids in treatment of the latter disease.

In summary, the book is concise, well-produced, up to date at time of publication and each chapter has a copious reference list. The index is adequate but finding one's way in the book would have been greatly assisted by the placing of authors' names at the top of even-numbered pages rather than merely repeating the not very distinctive running titles on every consecutive page. The book is of general interest to research workers in reproductive biology and to clinicians concerned with breast and endometrial cancer treatment. It is indispensable to laboratories involved in receptor assay.

L. G. Skinner